# Electronically assisted surveillance systems of healthcare-associated infections: a systematic review

**DOI:** 10.2807/1560-7917.ES.2020.25.2.1900321

**Published:** 2020-01-16

**Authors:** H Roel A Streefkerk, Roel PAJ Verkooijen, Wichor M Bramer, Henri A Verbrugh

**Affiliations:** 1 Erasmus University Medical Center (Erasmus MC), Rotterdam, the Netherlands; 2 Albert Schweitzer Hospital/Rivas group Beatrix hospital/Regionaal Laboratorium medische Microbiologie, Dordrecht/Gorinchem, the Netherlands; 3 Department of Medical Microbiology, University of Groningen, University Medical Center Groningen, Groningen, the Netherlands; 4 Medical Library, Erasmus MC University Medical Center Rotterdam, Rotterdam, the Netherlands

**Keywords:** surveillance, healthcare-associated infections, electronic, computer-assisted, Point prevalence survey

## Abstract

**Background:**

Surveillance of healthcare-associated infections (HAI) is the basis of each infection control programme and, in case of acute care hospitals, should ideally include all hospital wards, medical specialties as well as all types of HAI. Traditional surveillance is labour intensive and electronically assisted surveillance systems (EASS) hold the promise to increase efficiency.

**Objectives:**

To give insight in the performance characteristics of different approaches to EASS and the quality of the studies designed to evaluate them.

**Methods:**

In this systematic review, online databases were searched and studies that compared an EASS with a traditional surveillance method were included. Two different indicators were extracted from each study, one regarding the quality of design (including reporting efficiency) and one based on the performance (e.g. specificity and sensitivity) of the EASS presented.

**Results:**

A total of 78 studies were included. The majority of EASS (n = 72) consisted of an algorithm-based selection step followed by confirmatory assessment. The algorithms used different sets of variables. Only a minority (n = 7) of EASS were hospital-wide and designed to detect all types of HAI. Sensitivity of EASS was generally high (> 0.8), but specificity varied (0.37–1). Less than 20% (n = 14) of the studies presented data on the efficiency gains achieved.

**Conclusions:**

Electronically assisted surveillance of HAI has yet to reach a mature stage and to be used routinely in healthcare settings. We recommend that future studies on the development and implementation of EASS of HAI focus on thorough validation, reproducibility, standardised datasets and detailed information on efficiency.

## Introduction

Surveillance of healthcare-associated infections (HAI) entails the systematic collection of data on the presence of HAI, analysis and transformation of the data into information and sharing this information with those who can take action to prevent HAI. Surveillance with feedback is a key/core component for effective infection prevention and control in the strategies of both the World Health Organization (WHO) and the European Centre for Disease Prevention and Control (ECDC) [1,2]. Surveillance and epidemiology are the first criteria included in the minimum standard of practice of an infection preventionist (Association for Professionals in Infection Control and Epidemiology (APIC) 2016 professional and practice standards). They are also a core task according to the Dutch society of Hygiene and Prevention in Healthcare (VHIG 2014, professional profile). The Health and Social Care Act 2008 code of practice of the prevention and control of infections and related guidance (2015, United Kingdom) likewise states that there should be evidence of local surveillance and of the use of comparative data, where available, to monitor infection rates in healthcare settings. In 2005, legislation on mandatory reporting of HAI had already been enacted in 35 states of the United States. In 2008, the Centers for Medicare and Medicaid Services stopped additional payment to hospitals to cover for the costs of treating mediastinitis after coronary artery bypass graft surgery, because this HAI was deemed to be preventable by HAI surveillance and control*.* Likewise, surgical site infection (SSI) after specific orthopaedic procedures and bariatric surgery were not covered anymore. This change reflected the importance of HAI surveillance and prevention. Hospital accrediting authorities, including Joint Commission International, also recognise the importance of surveillance activities. 

Surveillance in its traditional format, in which every patient’s record is assessed on the presence of HAI, is labour intensive and can take up to 756 manhours for a point prevalence survey or an estimated 1.5 full-time equivalent (FTE) per 10,000 admissions [3,4]. Surveillance of HAI should ideally include all hospital wards, medical specialties as well as all types of HAI, such that fully informed, evidence-based decisions can be made to prioritise and structurally address the relevant infection issues of the particular healthcare setting. However, in the face of limited resources, available labour intensive manual surveillance systems have driven most healthcare centres to apply so-called targeted forms of surveillance that include only high risk wards and/or few types of medical procedures and/or only a few types of HAI.

Alternatively, efforts have been made to significantly improve the efficiency (i.e. reducing the time spent on surveillance while obtaining the same results) of HAI surveillance by applying information technologies to query data routinely available in hospital electronic databases. With the ongoing application of new information technologies in healthcare, the types and sizes of these electronic databases have been increasing over the last decades. It is, however, unclear what level of efficiency and accuracy (i.e. proportion of individuals truly positive and negative for an HAI) has been attained so far by applying these newer electronic information sources and technologies. Increasing the efficiency of surveillance can be achieved by introducing a computerised algorithm-based selection of high-risk patients followed by confirmatory assessment of the selected cases by the infection control practitioner (ICP) (semi-automated electronically assisted surveillance system (EASS)). Alternatively, surveillance can be based on the outcome of a computerised algorithm alone, without confirmation of individual cases by the ICP (fully automated EASS).

With this systematic review, we aim to give insight in the current status of EASS by assessing their performance (sensitivity and specificity) compared with traditional surveillance, the variables used in the electronic algorithms and the quality of the studies (including efficiency) that presented and evaluated them.

## Methods

### Inclusion of articles

In this systematic review, the Preferred Reporting Items for Systematic Reviews and Meta-Analyses (PRISMA) guidelines were followed [5]. The following online databases were searched on 10 January 2018: Embase, Medline Ovid, Cochrane, Web of Science, Scopus, CINAHL (EBSCOhost) and Google Scholar. The Embase search was the basis for the search method and then translated for use in other databases. All publications, except grey literature, in the past up to 10 January 2018 were included. The full description of the queries per database is provided in the supplementary material. The process of identification, screening and inclusion of articles for full text synthesis is consistent with the method described by Bramer et al. [6-8]. In brief, this method consists of a query in online databases, review of all the abstracts by two reviewers independently to screen for articles that probably meet the inclusion criteria (eligible) followed by full article review to decide whether an article is included based on the inclusion and exclusion criteria. Included were studies which systematically evaluated the performance of an electronically assisted or fully automated surveillance method against the gold standard (expert opinion of the ICPs based on the United States Centers for Disease Control and Prevention-based criteria for HAI [9]), and reported at least one performance metric (e.g. sensitivity, specificity). Exclusion criteria were potential incorporation bias, non-English language studies and poster or conference abstracts. 

### Data collection process

Using a standardised format, we abstracted the following reported information from each study: (i) year of publication, country and setting; (ii) the study type, population characteristics and sample size; (iii) the type of HAI (bloodstream infections (BSIs), lower respiratory tract infections (LRTIs), SSIs or urinary tract infections (UTIs)) targeted for surveillance, likewise the medical specialties or wards that were participating in the surveillance effort; (iv) the sensitivity, specificity, positive and negative predictive value, accuracy of the EASS; and (v) all variables that were used in their algorithms. Missing information was not checked with the authors.

### Algorithm categories

For each study, we assigned the respective algorithm used to one of the categories based on the variables included as presented in Table 1.

**Table 1:** Categorisation of the electronically assisted surveillance system’s algorithms based on the set of variables included

Category	Description
1	ICD coding only
2	Microbiology (bacterial, viral, fungal pathogens detected by culture, molecular or serological diagnostics)
3	Microbiology + antibiotic prescriptions
4	Microbiology + antibiotic prescriptions + clinical chemistry
5	Other (body temperature OR/AND judgement by physician OR/AND ventilator setting OR/AND fuzzy logic or natural language processing of clinical notes OR/AND risk factors, like indwelling catheters)

ICD: International Classification of Diseases.

### Overall quality and performance score

Two different scores were calculated based on the data extracted from each study, one regarding the reported design of the study and one regarding the performance of the EASS presented. First, to assess the quality of the design we scored six indicators that represent different aspects of the design of the study. As an example, for indicator 1, the rationale was as follows: an EASS should be evaluated in a set of patients that is independent of the set used to develop the automated method, as recommended by Govindan et al. in 2010 [10]. Thus when the study population consisted of a validation cohort and a separate development cohort this was considered better (5 points) than when the study population consisted of a development cohort only (1 point). Subsequently we calculated an overall quality score by adding up the points assigned for each of the six quality indicators presented in Table 2.

**Table 2:** Quality of study designs: indicators used to evaluate the level of quality of study designs investigating an electronically assisted surveillance system

Indicator – description	Points awarded
**Indicator 1** – validation and test cohort	
The study population consisted of a validation cohort and a separate development cohort	5
The study population consisted of a validation cohort	3
The study population consisted of a development cohort only	1
**Indicator 2** – reported context of study	
The observed prevalence or incidence of HAI in the healthcare institute was presented in the article	5
Prevalence or incidence of HAI was not presented in the article	0
**Indicator 3** – departments under surveillance for HAI	
EASS was hospital-wide and included all wards or departments	5
EASS was not hospital-wide but included > 2 departments or wards	3
EASS was targeted to one department or ward only	1
**Indicator 4** – types of HAI under surveillance	
EASS targeted all types of HAI	5
EASS targeted > 2 types of HAI	3
EASS targeted one or two types of HAI only	1
**Indicator 5** – performance characteristics reported	
Sensitivity, specificity and other performance characteristics of the EASS algorithm were presented	5
Only sensitivity and specificity were presented	3
Only sensitivity was presented	1
**Indicator 6** – efficiency reported	
Time reduction was presented quantitatively	5
Workload reduction was presented	3
No data on workload or time reduction presented	1

HAI: healthcare-associated infection; EASS: electronically assisted surveillance system.

The red colour represents poor quality, the orange intermediate quality and green indicates good quality.

Secondly, for each study we calculated an overall performance score of the EASS presented in the study by multiplying its published sensitivity and specificity. This overall performance score is primarily based on the sensitivity and specificity to detect all types of HAI. If not all types of HAI were included we calculated an overall performance score based on the means of the sensitivities and specificities specified for the types of HAI that were included in the study.

### Ethical statement

Ethical approval was not applicable for this review of published literature.

## Results

**Figure 1:**
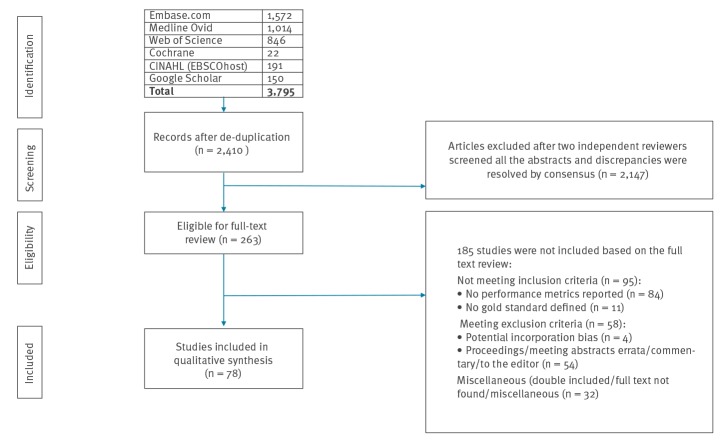
A flowchart of the inclusion process of studies used in the systematic review (adjusted from the PRISMA 2009 flowchart)

The online databases search identified 2,410 records after de-duplication, of which 78 studies [3,4,11-86] were included in this systematic review (Figure 1).

PRISMA: Preferred Reporting Items for Systematic Reviews and Meta-Analyses.

### Timeline and geographical distribution

**Figure 2:**
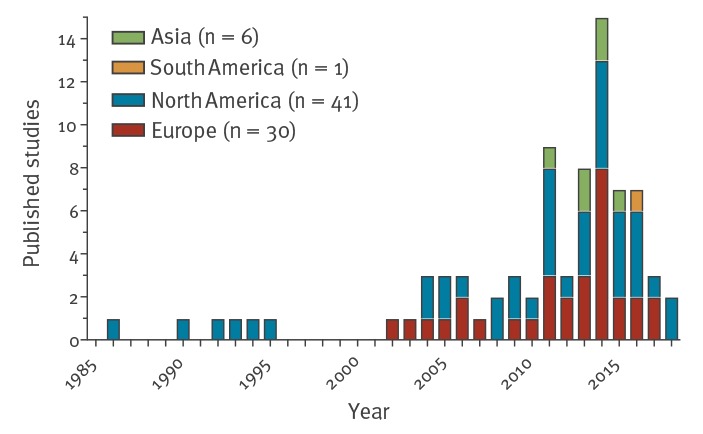
Published studies on electronically assisted surveillance of healthcare-associated infections by year of publication and by region of the world (n = 78)

Of the 78 identified studies, 30 were from Europe, six from Asia, one from South America and the remaining 41 from North America. The first articles about EASS of HAI originated in North America and were published in the 1980s and 1990s. Thereafter, their number increased gradually, involving European centres since the turn of the century, and, more recently centres in other parts of the world; a peak in the annual number of published articles was reached in 2014 (Figure 2).

In the earliest identified study, in 1986, Evans et al. [30] presented the results of HAI surveillance through their Health Evaluation through Logical Processing (HELP) system at the Latter Day Saints hospital, Salt Lake City, Utah. These pioneers showed that more HAI were found with this EASS, while its execution required only 35% of the time. In follow-up studies HELP was used to evaluate algorithms to predict infections even before onset of symptoms [29]. It was not until the year 2003 that the first European study was published describing the performance of an EASS; this EASS was implemented in 1998 in a university affiliated teaching hospital in France [11]. From 2000 to 2009 predominantly American and European research groups presented their EASS in the international peer-reviewed literature. In the year 2006, Brossette et al. [4] published the first results of their EASS that used a laboratory-based algorithm, called Nosocomial Infection Marker (NIM), to help identify patients with HAI. This software was the first to become commercially available. From 2010 on, the numbers of publications increased and peaked in 2014. Contributions originating from South America [56] and Asia [3,24,50,67,68,77] showed that by that time EASS of HAI had adopters worldwide.

### Fully- vs semi-automated surveillance

Only six of the 78 studies (8%) present a fully automated surveillance method [20,27,52,62,65,69], with sensitivities ranging from 0.6 to 0.94 for UTIs and ventricular drain infections respectively.

### Quality

As for indicator 1 of the overall quality score (Table 2), 18 of the 78 (23%) articles included in this review used a development and separate validation cohort. The majority of articles (53 of 78) clearly presented the point prevalence or incidence of the HAI in the population they studied (quality indicator 2, Table 2). The EASS was hospital-wide (maximum points for quality indicator 3, Table 2) and included all types of HAI (maximum points for quality indicator 4, Table 2 with at least the ‘big four’ types of HAI, i.e. BSIs, LRTIs, UTIs and SSIs) in nine of the 78 studies. Just under 40% (31/78) of the studies presented performance characteristics other than only sensitivity and specificity (maximum points for indicator 5). Less than 20% (14/78) of the studies presented the actual time reduction that was achieved by introducing an EASS for HAI (maximum points for indicator 6, Table 2). None of the articles attained the maximum score (all six indicators ‘green or maximum points appointed’) and, interestingly, the overall quality score did not seem to improve over the years (supplementary material Table 1).

### Performance of electronically assisted surveillance systems per type of healthcare-associated infection

EASS were most extensively developed and applied in intensive care unit (ICU) settings (44 of the 78 articles included in this review); 20 articles describe an EASS that was developed exclusively for the ICU and targeted the ICU’s two most common HAI (ventilator associated pneumonia (VAP) and central line associated BSIs (CLABSI)) [15,18,27,28,32,33,38,40,42,43,51,52,55,57,61,62,73,75,78,81]. Other studies focused on one particular type of HAI (one or more of the ‘big four’: LRTIs, BSIs, SSIs and UTIs) and aimed to facilitate its detection and registration hospital-wide [3,4,13,21,23-25,29,30,50,58,59,63,66-68,74,79,80,83,86]. Among these a few research groups tried to develop the ‘one algorithm to find them all’ [3,4,21,23,24,29,30], i.e. one algorithm that can be used to detect all types of HAI in all departments and medical specialties. The studies that aimed to achieve this, present sensitivities ranging from 0.78 to 0.99. Du et al. [3] describe an excellently performing algorithm (sensitivity 0.99, specificity 0.93) to detect HAI in real time. Interestingly, they presented a decision support system to aid the ICP professional in making the final ‘HAI present or not’- decision. Doing so, they claim to have increased the overall specificity of their surveillance system to 0.99. A downside to their approach was a decrease of sensitivity to 0.94. This was due to the fact that the information provided by the support software was sometimes not sufficient for final confirmation of the presence of a HAI or no HAI.

### Algorithms and parameters used in electronically assisted surveillance system

In their review ‘Surveillance and use of computers in hospital infection control’ Wenzel and Streed [87] presented a timeline of the evolution of surveillance of infectious diseases including healthcare-associated ones, from 1532 to 1989, pointing out key figures and events in its history. As they elaborated on the different elements of surveillance, the authors eventually addressed how the application of computers could help infection control with their surveillance activities and add to their accuracy and probably to efficiency as well. In 2002 Peterson and Brossette [88] addressed the importance of clinical microbiological laboratories and the clinical microbiologist in HAI control. They stated that laboratory-based surveillance has the advantage of measuring hospital-wide occurrences from a single, central data collection point. These microbiological data could be used in a hospital-wide EASS of HAI. In 2008, Leal et al. [89] reviewed 24 articles in which microbiological data constituted the predominant variable used in electronic surveillance systems for HAI. Among those reviewed by Leal et al., six studies reported that HAI could be detected using microbiology data alone with reasonable to good overall sensitivity (range: 0.63–0.91) and excellent specificity (range: 0.87 to > 0.99). According to the Leal et al. review, seven studies using only administrative data including discharge coding (International Classification of Diseases (ICD), 9th edition, Clinical Modification) and pharmacy data reported good sensitivity (range: 0.59–0.96) and excellent specificity (range: 0.95 to > 0.99) in detecting HAI. Moreover, six studies using both laboratory and administrative data reported a higher sensitivity (range: 0.71–0.94) but lower specificity (range: 0.47 to > 0.99) compared with the use of either alone. In 2013, Freeman et al. [90] performed a systematic review in which they categorised the EASS in multisource (including microbiological data) (n = 37), multisource (excluding microbiological data) (n = 4) and single source systems (n = 3). Of these 44 articles included for review, 21 were validation studies. There was no difference in the performance of multisource systems excluding or including microbiology. Of note, the single source validation studies, used natural language processing techniques to extract information from the radiology reports and electronic health record to detect LRTI and BSI respectively [33,91]. Based on the results of their review in 2014 de Bruin et al. [92] concluded that driven by the increased availability of electronic patient data, EASS tend to use more data sources. This is making systems more sensitive yet less specific, but also allows systems to be tailored to the needs of healthcare institutes’ surveillance programmes. The findings of Cato’s review [93], in 2015, suggest that the majority of EASS for HAI surveillance are using standard definitions of HAI, but the lack of standardised use of data formats, denominator, and external validation in these systems reduces the reliability of their findings.

### Microbiological data

**Figure 3:**
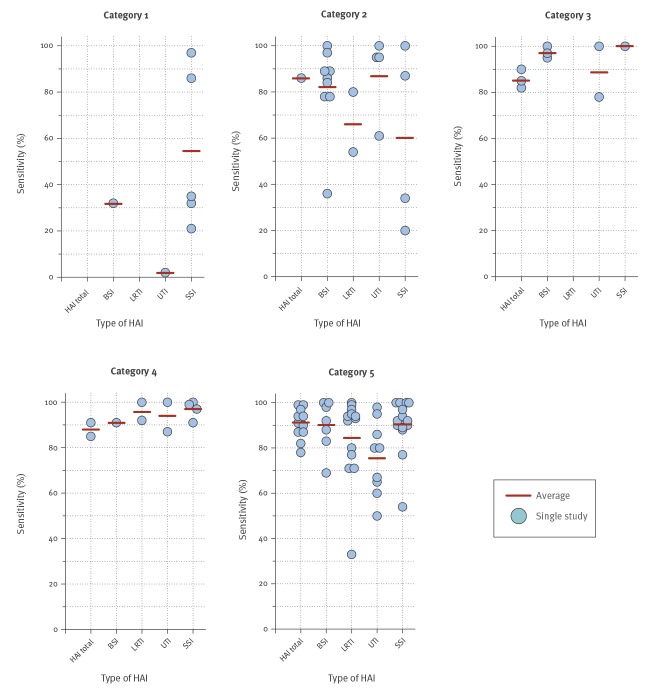
Sensitivity of electronically assisted surveillance systems by category of algorithm used and by type of healthcare-associated infection (n = 78)

BSI: bloodstream infections; HAI: healthcare-associated infection; LRTI: lower respiratory tract infections; SSI: surgical site infections; UTI: urinary tract infections.

The most prevalent variable included in EASS is the microbiological examination (present in at least one of the algorithms presented in 61 of the 78 articles). It is a crucial variable in algorithms for surveillance of BSI. Good sensitivities have been achieved using the results of microbiological examinations as the sole variable (Figure 3). In other types of HAI (UTI, SSI and LRTI) the sensitivity of algorithms that use microbiological examination alone is lower. As a consequence of the definitions used as ‘gold standard’, the microbiological examination variable is often combined with clinical characteristics (Figure 3). Although the microbiology variable seems straightforward, it can mean several things, i.e. the presence of bacteriological examinations, which use either culture, serological or molecular techniques or all of these. The presence and quality of the microbiological examination highly depends on the availability of the service – often lacking in low income settings –, and on the adherence to diagnostic protocols. Finally, a negative microbiological examination does not unequivocally exclude the presence an infectious disease.

Category 1: international classification of diseases coding only; category 2: microbiology (bacterial, viral, fungal pathogens detected by culture, molecular or serological diagnostics); category 3: microbiology + antibiotic prescriptions; category 4: microbiology + antibiotic prescriptions + clinical chemistry; category 5: other types of algorithms (using parameters like body temperature OR/AND judgement by physician OR/AND ventilator setting OR/AND fuzzy logic or natural language processing of clinical notes OR/AND risk factors, like indwelling catheters). The dots represent the sensitivities for each individual study per type of HAI, the red lines represent the average sensitivities.

### Chemistry

Clinical chemistry evaluation of biomarkers for infections are routinely performed in most clinical settings. Leukocyte counts and C-reactive protein (CRP) are the variables of choice in different algorithms made for HAI surveillance, but they are always, in all 19 of 78 studies that use this variable, used in combination with other parameters [3,26,35,36,39,42,43,45,46,49,56,64,65,70,72,73,75,82,87].

### Antibiotics

Following microbiological data, antibiotic prescriptions data are the second most used variable in EASS algorithms (38 of 78 studies) and, when not combined with microbiological data, most often found in combination with ICD coding [12,16,36,56].

### International Classification of Diseases

The sensitivity of ICD-coding (9th or 10th edition) is generally low, in five of the seven studies that use this as the sole variable of the algorithm [11,39,54,70,77,78,83]. The two studies with sensitivities of 0.86 and 0.97 focused on SSI after coronary artery bypass graft surgery and total joint replacement [39,54]. ICD-coding has not been studied for all types of HAI surveillance nor for LRTI surveillance (Figure 2).

### Electronically assisted surveillance system per type of healthcare-associated infection

#### Bloodstream infections (including central line associated)

Twenty-three publications (Table 3) described the performance of an EASS to detect BSI. Sensitivity ranged from 0.32 to 1.0, specificity from 0.37 to 1.

**Table 3:** Performance characteristics of electronically assisted surveillance systems for surveillance of bloodstream infection (including central line associated infection) (n = 23 studies)

Ref.	First author	Algorithm category	Year of publication	Sensitivity	Specificity	PPV	NPV	Accuracy^a^	Concordance
[68]	Tseng	Other	2013	NA	NA	NA	NA	0.94	NA
[82]	Leal	Microbiology	2016	NA	NA	NA	NA	NA	0.97
[18]	Bouzbid	Other	2011	1	0.37	0.1	1	NA	NA
[28]	De Bus	Other	2014	1	1	NA	NA	NA	NA
[58]	Redder	Microbiology + antibiotics	2015	1	1	0.88	1	NA	NA
[73]	Venable	Microbiology	2013	1	0.92	NA	NA	NA	NA
[67]	Tseng	Other	2015	0.98	0.99	0.96	1	NA	NA
[75]	Woeltje	Microbiology + antibiotics	2008	0.97	0.44	0.15	0.99	NA	NA
[66]	Trick	Microbiology	2004	0.97	0.73	NA	NA	NA	NA
[76]	Woeltje	Microbiology + antibiotics	2011	0.95	0.98	0.9	0.99	NA	NA
[40]	Kaiser	Other	2014	0.92	1	1	1	NA	NA
[63]	Streefkerk	Microbiology + antibiotics + chemistry	2014	0.91	NA	NA	NA	NA	NA
[59]	Ridgway	Microbiology	2016	0.89	1	NA	1	NA	NA
[17]	Bouam	Microbiology	2003	0.89	0.75	0.63	0.93	NA	NA
[35]	Henry	Other	2013	0.88	0.92	NA	NA	NA	NA
[4]	Brossette	Microbiology	2006	0.86	1	NA	NA	NA	NA
[32]	Graham	Microbiology	2004	0,84	0.99	0.84	0.99	NA	NA
[86]	Streefkerk	Microbiology + antibiotics + chemistry	2016	0.83	NA	NA	NA	NA	NA
[14]	Bellini	Microbiology	2007	0.78	0.93	NA	NA	NA	NA
[61]	Stamm	Microbiology	2012	0.78	NA	0.5	NA	NA	NA
[13]	Bearman	Other	2010	0.69	0.88	0.05	NA	NA	NA
[80]	Gubbels	Microbiology	2017	0.36	0.99	NA	NA	NA	NA
[78]	Bond	ICD codes	2016	0.32	NA	NA	NA	NA	NA

ICD: International Classification of Diseases; HAI: healthcare-associated infection; NA: data not available; NPV: negative predictive value; PPV: positive predictive value; ref.: reference.

^a^ Accuracy is calculated as: (TP+TN) / (TP+TN+FP+FN) where TP is the true positive (i.e. the number of individuals correctly identified as positive for a given HAI), FP is the false positive (i.e. the number of individuals incorrectly identified as positive for the given HAI), TN is the true negative (i.e. the number of individuals correctly identified as negative for the given HAI) and FN is the false negative (i.e. the number of individuals incorrectly identified as negative for the given HAI).

In 2017, Gubbels et al. presented the results of an automated nationwide survey, using the Danish Microbiology Database and the Danish National Patient Registry. National trends showed an increase in hospital-acquired bacteraemia (HAIBA) cases between 2010 and 2014. Although comparison of their HAIBA algorithm with the results of point prevalence surveys showed a sensitivity of 0.36 they concluded that given the many advantages of automated surveillance, the HAIBA algorithm allows monitoring of HAIBA across the healthcare system, supports prioritising preventive measures, and holds promise for evaluating interventions [80]. Another approach to automated surveillance of BSI is the reuse of the ICD-coding system. Unfortunately the performance to detect BSI in the paediatric ICU was shown to be very poor (sensitivity of 0.32), as Bond et al. showed [78]. In contrast, one adult ICU-tailored algorithm showed excellent performance characteristics (sensitivity and specificity of 1) [28]. The study was conducted at the 14-bed medical ICU (MICU) and the 22-bed surgical ICU (SICU) of Ghent University Hospital (1,050 beds), where Computer-based Surveillance and Alerting of infections Antimicrobial Resistance and Antibiotic consumption (COSARA) software has been available since 2010. With COSARA, all infection-related data from the various electronic patient records are integrated and presented to the treating ICU physician by means of a continuously updated clinical dash-board. This includes a graphical display of current and past antibiotic treatments as a timeline, and provides direct links to a real-time copy of the various source records. The graphical interface allows episodes of antibiotic treatment to be labelled according to a predefined list of indications and diagnoses, and linked with microbiological culture results [28]. Surveillance for CLABSIs by infection control practitioners is often limited to ICUs. Woeltje et al. applied an automated surveillance system for CLABSI outside the ICU. Is this study they evaluated the performance of 12 different rule sets for the detection of CLABSI. The best-performing rule set had an overall sensitivity of 0.95, specificity of 0.97, positive predictive value of 0.90, and negative predictive value of 0.99 compared with intensive manual surveillance. The method offered the possibility of performing acceptably good surveillance in areas where resources do not allow for traditional manual surveillance [76]. This hospital-wide approach generally results in lower sensitivities [4,13,63]. However, Tseng et al. showed their hospital-wide EASS system performed very well in a 2,200-bed teaching hospital, with regard to sensitivity (0.98), specificity (0.99), positive predictive value (0.96), and negative predictive value (1) compared with their reference standard, which, remarkably, was a continuous manual, hospital-wide surveillance for BSI, including CLABSI, operating since 1981 [67].

#### Lower respiratory tract infections (including ventilator-associated pneumonia)

Sixteen publications (Table 4) from 2005 to 2018 presented the performance of an EASS to detect LRTIs, including hospital-acquired pneumonia (HAP), mostly VAP. Sensitivity ranged from 0.33 to 1.0 and specificity from 0.58 to 1.0. The majority of the studies (14/16) addressed the performance of an EASS to detect VAP in an ICU setting, including two neonatal ICUs. Because of the complexity of the definitions, ventilator-associated events (VAE), ventilator-associated conditions (VAC) or VAP, comparing the studies’ methods and results is difficult.

**Table 4:** Performance characteristics of electronically assisted surveillance systems for surveillance of lower respiratory tract infection (including ventilator associated infection) (n = 16 studies)

Ref.	First author	Algorithm category	Year of publication	Sensitivity	Specificity	PPV	NPV
[51]	Mann	Other	2015	1	1	1	1
[81]	Hebert	Microbiology + antibiotics + chemistry	2018	1	NA	NA	NA
[18]	Bouzbid	Other	2011	0.99	0.58	0.22	1
[26]	Claridge	Other	2009	0.97	1	NA	NA
[42]	Klompas	Other	2008	0.95	1	NA	NA
[86]	Streefkerk	Microbiology + antibiotics + chemistry	2016	0.94	NA	NA	NA
[62]	Stevens	Other	2014	0.94	NA	1	NA
[55]	Nuckchady	Other	2015	0.93	0.99	0.95	0.98
[40]	Kaiser	Other	2014	0.92	1	1	1
[63]	Streefkerk	Microbiology + antibiotics + chemistry	2014	0.92	NA	NA	NA
[35]	FitzHenry	Other	2013	0.8	0.9	NA	NA
[28]	De Bus	Other	2014	0.77	0.99	NA	NA
[33]	Haas	Other	2005	0.71	0.95	0.08	1
[52]	Mendonca	Other	2005	0.71	0.99	0.075	NA
[61]	Stamm	Microbiology	2012	0.54	NA	0.25	NA
[43]	Klouwenberg	Other	2014	0.33	NA	0.25	NA

NA: data not available; NPV: negative predictive value; PPV: positive predictive value; ref.: reference.

#### Urinary tract infections (including catheter-associated urinary tract infections)

UTI is one of the ‘big four’ and is third, behind SSI and LRTI, in HAI prevalence in the Netherlands (2017) [94]. Internationally 18 publications (Table 5) presented the performance of an EASS to detect catheter-associated (CA)UTI. Reported sensitivities ranged from 0.02 to 1.0, whereas specificities ranged from 0.59 to 1.0. There are several reasons for the low sensitivity described in the report by Condell et al., including laboratory results being unavailable at the time of the survey, the results considered clinically irrelevant by the surveyor due to an indwelling urinary catheter or lack of clinical signs of infection. UTIs being considered hospital-acquired in ‘the gold standard’ even though the first sample was taken within 48 hours of admission also compromised sensitivity [79]. ICD-10 codes for UTI surveillance were shown to perform poorly (sensitivity 0.02) and were only studied in one study [83]. On the other hand a urine culture as the only parameter proved to perform well [38,50].

**Table 5:** Performance characteristics of electronically assisted surveillance systems for surveillance of urinary tract infection (including catheter-associated infection) (n = 18 studies)

Ref.	First author	Algorithm category	Year of publication	Sensitivity	Specificity	PPV	NPV
[50]	Lo	Microbiology + antibiotics	2013	1	0.95	NA	NA
[38]	Hsu	Microbiology + antibiotics + chemistry	2015	1	1	NA	NA
[73]	Venable	Microbiology	2013	1	0.97	NA	NA
[18]	Bouzbid	Other	2011	0.98	0.59	0.183	1
[17]	Bouam	Microbiology	2003	0.95	1	1	0.95
[4]	Brossette	Microbiology	2006	0.95	1	NA	NA
[35]	Henry	Other	2013	0.95	0.8	NA	NA
[63]	Streefkerk	Microbiology + antibiotics + chemistry	2014	0.87	NA	NA	NA
[25]	Choudhuri	Other	2011	0.86	0.94	0.85	0.94
[28]	De Bus	Other	2014	0.8	0.99	NA	NA
[74]	Wald	Other	2014	0.8	0.99	0.69	0.99
[58]	Redder	Microbiology + antibiotics	2015	0.78	0.93	0.88	0.87
[86]	Streefkerk	Microbiology + antibiotics + chemistry	2016	0.67	NA	NA	NA
[20]	Branch-Elliman	Other	2015	0.65	1	0.54	1
[62]	Stamm	Microbiology	2012	0.61	NA	0.47	NA
[65]	Tanushi	Other	2014	0.6	0.99	NA	0.98
[79]	Condell	Other	2016	0.5	0.94	NA	NA
[83]	Marra	ICD codes	2017	0.02	NA	NA	NA

ICD: International Classification of Diseases; NA: data not available; NPV: negative predictive value; PPV: positive predictive value; ref.: reference.

#### Surgical site infections

Not surprisingly, the majority of publications, 29 in total, presented the performance of an EASS to detect SSI (Table 6). Different fields of surgery are targeted, from neurosurgery to total hip replacements and different algorithms with sensitivities ranging from 0.02 to 1.0 and specificities ranging from 0.59 to 1.0 have been explored. Administrative data (ICD-9 admission and discharge coding and claims data) generally performed poorly when used as the only variable for case finding [37,46,54,70,77].

**Table 6:** Performance characteristics of electronically assisted surveillance systems for surveillance of surgical site infection (n = 29 studies)

Ref.	First author	Algorithm category	Year of publication	Sensitivity	Specificity	PPV	NPV	Accuracy
[11]	Apte	ICD codes	2011	0.86	NA	NA	NA	NA
[12]	Baker	Other	1995	0.89	0.95	0.53	NA	NA
[16]	Bolon	Other	2009	1	NA	0.07	1	NA
[19]	Branch-Elliman	Other	2014	0.97	0.98	NA	NA	NA
[4]	Brossette	Microbiology	2006	1	0.6	NA	NA	NA
[23]	Chalfine	Microbiology	2005	0.84	1	NA	NA	NA
[31]	Gerbier-Colomban	Other	2012	0.92	0.86	NA	NA	NA
[34]	Hautemanière	Other	2013	0.54	0.95	0.74	0.88	NA
[35]	Henry	Other	2013	0.77	0.63	NA	NA	NA
[37]	Hollenbeak	Microbiology	2011	0.2	0.96	NA	NA	0.89
[39]	Inacio	ICD codes	2011	0.97	0.92	NA	NA	NA
[41]	King	Other	2014	0.9	0.94	NA	NA	NA
[44]	Knepper	Other	2013	1	0.88	NA	NA	NA
[45]	Knepper	Other	2014	0.94	0.88	NA	NA	NA
[46]	Kulaylat	Microbiology	2016	0.37	1	0.72	0.99	NA
[47]	Leclere	Other	2014	0.90	0.98	0.25	1	NA
[49]	Leth	Other	2010	0.90	0.98	0.54	1	NA
[53]	Michelson	Other	2014	1	NA	NA	NA	NA
[54]	Moro	ICD codes	2004	0.21	NA	NA	NA	NA
[63]	Streefkerk	Microbiology + antibiotics + chemistry	2014	0.91	NA	NA	NA	NA
[69]	van Mourik	Microbiology + antibiotics + chemistry	2011	0.99	0.88	0.57	1	NA
[70]	van Mourik	ICD codes	2013	0.32	NA	0.35	NA	NA
[71]	van Mourik	Microbiology + antibiotics + chemistry	2012	1	NA	0.59	NA	NA
[72]	van Mourik	Microbiology + antibiotics + chemistry	2015	0.97	NA	0.47	NA	NA
[77]	Yu	ICD codes	2014	0.35	0.97	0.19	0.99	NA
[56]	Perdiz	Other	2016	0.88	1	1	1	NA
[86]	Streefkerk	Microbiology + antibiotics + chemistry	2016	1	NA	NA	NA	NA
[84]	Pindijck	Other	2018	0.92	0.57	NA	NA	NA
[85]	Sips	Microbiology + antibiotics	2017	1	NA	0.68	NA	NA

ICD: International Classification of Diseases; NA: data not available; NPV: negative predictive value; PPV: positive predictive value; ref.: reference.

Interestingly a Michigan study by Baker et al. showed a 0.89 sensitivity for ICD-9 codes when these were solely used to determine post-caesarean section SSIs. Combining ICD-9 with specific post-operative antibiotics increased the specificity of the case finding algorithm to 0.95 and resulted in a ca 90% workload reduction [12]. Fifteen years later in Denmark, comparable performance characteristics were found for in-hospital and post-discharge surveillance of post-caesarean section SSIs using ICD-10 discharge codes in combination with relevant antibiotics and culture data [49].

Adding microbiology and radiographic data (culture, magnetic resonance imaging ordered) as variables of case finding algorithms resulted in a positive predictive value of 0.97, although at the cost of sensitivity (0.48) [19]. A text mining algorithm as presented by Michelson et al. found all of the 22 SSIs detected by traditional hospital-based surveillance, along with an additional 37 SSIs not detected by traditional surveillance [53].

### Quality and performance

**Figure 4:**
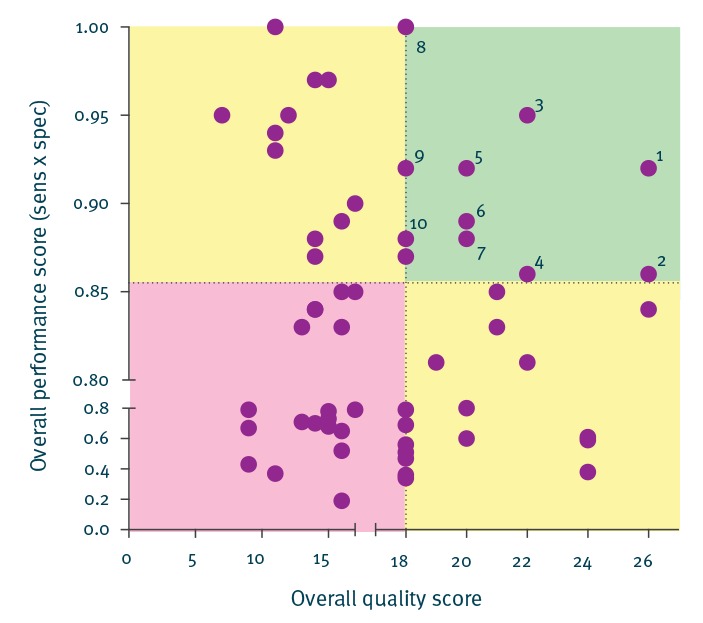
Distribution of overall quality score of studies on electronically assisted surveillance systems vs the overall performance score of the electronically assisted surveillance system reported in these studies (n = 78)

The quality of the 78 studies included in this review varied from an overall quality score of 5 to 26. Likewise, the overall performance score of the EASS developed and used for HAI surveillance varied widely among the studies, with overall performance scores ranging from a low 0.2 to the maximum of 1.0. When quality of study design was plotted against the level of performance of the EASS there seemed to be a, albeit weak, negative correlation, suggesting that the best designed studies tended to yield lower performance scores or vice versa, that the studies reporting very high performance scores of their EASS were generally less well designed (Figure 4).

Sens: sensitivity; spec: specificity.

In the upper right quadrant the 10 best studies are ranked from 1 to 10 (see Table 7 for more detail).

Based on this view of the overall quality and performance score we selected the 10 best studies (labelled in the figure) that showed the highest overall quality scores and also yielded a high overall performance score ≥ 0.85 (Figure 4 upper right quadrant). They, together, constitute a reference or can be considered a benchmark, for EASS development. Interestingly all these studies were reported only recently (2013–2016), indicating that some progress has been made in designing and evaluating EASS (Table 7).

**Table 7:** Summary of the 10 best studies on electronically assisted surveillance systems included in the systematic review, ordered based on their overall quality score

**Ref.**	**First author**	**Year**	**Overall quality score**	**Overall performance score**	**Hospital-wide**	**All types of HAI**	**ICP reviews preselected cases only**	**Time reduction achieved**	Dataset used							
									**Microbiology**		**Microbiology + clinical chemistry**	**Microbiology + antibiotics**		**Clinical characteristics**		**ICD coding**
[3]	Du M	2014	26	0.92	Yes	Yes	Yes	Yes		Yes	Yes		Yes		Yes	No
[27]	De Bruin JS	2013	26	0.86	No	Yes	No	Yes		Yes	Yes		Yes		Yes	No
[50]	Lo Y	2013	22	0.95	Yes	No	Yes	No		Yes	No		Yes		Yes	No
[58]	Redder JD	2015	22	0.86	Yes	No	Yes	No		Yes	No		Yes		Yes	No
[40]	Kaiser AM	2014	20	0.92	No	No	Yes	Yes		No	No		Yes		Yes	No
[59]	Ridgway JP	2016	20	0.89	Yes	No	Yes	No		Yes	No		No		No	No
[45]	Knepper BC	2013	20	0.88	No	No	Yes	No		Yes	Yes		No		No	Yes
[51]	Mann T	2015	18	1.00	No	No	No	Yes		No	No		No		Yes	No
[55]	Nuckchady D	2015	18	0.92	No	No	No	Yes		No	Yes		No		Yes	No
[47]	Leclère B	2014	18	0.88	No	No	Yes	No		Yes	No		No		No	Yes

ICD: International Classification of Diseases; ICP: infection control practitioner; HAI: healthcare-associated infection; ref.: reference.

The cells in this table are green when a finding is present and red when absent.

Interestingly, the majority of the 10 best EASS presented in Table 7 used a two-step procedure, first a selection step based on an automated algorithm, followed by step in which a confirmatory assessment of the selected cases by the ICP is performed. The monitoring of nosocomial infections (MONI) software on the ICU, the MONI-ICU system [27], however, achieved a high specificity of the EASS for all types of HAI based on an automated selection step only. This was achieved in a university hospital ICU by building a system consisting of fuzzy logic sets, which used a rich dataset including clinical, laboratory and nursing data from the patient data management systems in operation at the ICU wards on a daily basis. Du et al. [3] showed that based on a dataset including microbiology, clinical chemistry, antibiotics prescriptions and clinical characteristics, it is possible to perform continuous hospital-wide all type HAI surveillance with an EASS. Of note, they stated that their hospital has the most advanced computer information system in China, including an integrated hospital information system (IHIS). When documented in the electronic patient record clinical characteristics, like body temperature or the presence of invasive devices, improve sensitivity and specificity of EASS. The data in Table 7 suggests that richer datasets lead to better performing EASS. Remarkably only five articles presented the time reduction achieved by using an EASS.

## Conclusions

Ideally, surveillance of HAIs comprises all departments, medical specialties and includes all types of HAI, because only then fully informed decisions can be made to prioritise and structurally address the relevant infection issues of the particular healthcare setting. In this review of the literature we found that very few EASS are ‘all types, hospital-wide’, although performance characteristics of these systems are generally good. The absence of traditional hospital-wide surveillance of HAI by the infection preventionist, which can be used as gold standard for validation can be one cause. On the other hand, the fact that most studies have been performed in an ICU setting implicates that hospital-wide electronic health records or data warehouses are still not ready to be used for decision support and surveillance.

Because of limited resources and the labour intensity of manual surveillance systems, efforts have been made to improve the efficiency of HAI surveillance by applying information technologies to query electronic datasets. However, we found that in literature, less than 20% of the studies present the actual time reduction that was achieved by introducing an EASS for HAI. Oher quality indicators, most importantly using different populations for development, testing and validation, are also lacking. Although sensitivity of EASS is generally high the specificity is variable, needing a confirmatory computer-assisted assessment by the infection preventionist and decision support. The parameters used in the algorithms studied vary considerably, do not have a standardised format nor are readily available in all hospital settings around the globe.

Although PRISMA guidelines were followed, this review has some limitations. Only English literature and no grey literature were considered for inclusion. The scoring system that was developed for and used in this study to assess the quality of the included studies’ design was not prior validated.

In summary, thus far computer-assisted surveillance of HAI has not reached a mature stage, it is yet to be used routinely in most healthcare settings; we are learning, but we have not yet mastered the art. Although progress is being made towards a digital infrastructure for the learning health system [95], it is, in our opinion, not likely that EASS of HAI will be implemented globally within the next decade. A data-driven and decision-supported healthcare system, including infection control surveillance, requires next generation electronic health records systems [96], clinical ownership and a good and close working relationship between infection control professionals and medical information specialists [97].

We recommend that future studies on the development and implementation of EASS of HAI focus on thorough validation, reproducibility in different hospital-settings of the algorithm and standardised datasets and present detailed information on efficiency. This information, together with the continuous focus on the importance of surveillance of HAI, is needed to convince healthcare providers, professionals and boards of directors, to invest in EASS, in the future of infection control. 

## Note

Registration number: this systematic review was not à priori registered.

## Supplementary Data

1231000 Supplementary Material
