# Supplementary material for: Electronically assisted surveillance systems of healthcare-associated infections: a systematic review
Source: Euro Surveill. 2020 Jan 16;25(2):1900321. doi: 10.2807/1560-7917.ES.2020.25.2.1900321 (PMC6976884; doi:10.2807/1560-7917.ES.2020.25.2.1900321)
Supplement: Supplementary Material [file 1900321_STREEFKERK_Supplementary_Material.pdf]

Supplementary material

H.R.A. Streefkerk, R.P.A.J. Verkooijen, W.M. Bramer, H.A. Verbrugh

"This supplementary material is hosted by *Eurosurveillance* as supporting information alongside the article “Electronically assisted surveillance systems (EASS) of healthcare-associated infections: a systematic review”, on behalf of the authors, who remain responsible for the accuracy and appropriateness of the content. The same standards for ethics, copyright, attributions and permissions as for the article apply. Supplements are not edited by *Eurosurveillance* and the journal is not responsible for the maintenance of any links or email addresses provided therein."

Inclusion of articles

In this systematic review the Preferred Reporting Items for Systematic Reviews and Meta-Analyses (PRISMA) guidelines were followed [1]. The process of identification, screening and inclusion of articles for full text synthesis is consistent with the method described by Bramer et al. [2, 3].

Information sources and search

The following online databases were searched on April 22, 2016 and on January 10 2018: Embase.com, Medline Ovid, Cochrane, Web of science, PubMed publisher, Scopus, CINAHL (EBSCOhost) and Google scholar Search. The search was programmed by WB and HRAS. The following search criteria were used:

Embase.com

('disease surveillance'/de OR (surveillance OR point-prevalence* OR incidence-rate*):ab,ti) AND ('hospital infection'/exp OR 'cross infection'/de OR 'surgical infection'/exp OR 'catheter infection'/de OR 'ventilator associated pneumonia'/de OR 'hospital acquired pneumonia'/de OR 'bloodstream infection'/exp OR (((hospital* OR nosocomial* OR ward OR healthcare OR health-care OR blood-stream* OR bloodstream* OR surg* OR operative* OR postop* OR postsurg* OR catheter* OR ventilator* OR ICU OR NICU OR PICU OR intensive-care OR cross) NEAR/6 (infection*)) OR ((hospital* OR ventilator*) NEXT/1 (acquire* OR associat*) NEXT/1 (pneumon* OR event*)) OR hai OR hais OR hcai OR hcais OR ssi OR vap OR hap OR vaps OR haps OR clabsi):ab,ti) AND ('automation'/exp OR 'electronics'/exp OR 'computer program'/exp OR 'information processing'/de OR 'bioinformatics'/de OR 'computer analysis'/de OR 'computer model'/de OR 'computer prediction'/de OR 'information system'/exp OR 'medical informatics'/exp OR 'electronic medical record'/exp OR 'information processing device'/de OR (automat* OR computer* OR electronic* OR software* OR data-mining OR ((surveillance) NEXT/1 (tool*)) OR repositor* OR information-system* OR informatics* OR bioinformatics* OR (electronic* NEAR/3 record*)):ab,ti) AND [english]/lim

Medline Ovid

((surveillance OR point-prevalence* OR incidence-rate*).ab,ti.) AND (exp "Cross Infection"/ OR "Surgical Wound Infection"/ OR "Catheter-Related Infections"/ OR (((hospital* OR nosocomial* OR ward OR healthcare OR health-care OR blood-stream* OR bloodstream* OR surg* OR operative* OR postop* OR postsurg* OR catheter* OR ventilator* OR ICU OR NICU OR PICU OR intensive-care OR cross) ADJ6 (infection*)) OR ((hospital* OR ventilator*) ADJ (acquire* OR associat*) ADJ (pneumon* OR event*)) OR hai OR hais OR hcai OR hcais OR ssi OR vap OR hap OR vaps OR haps OR clabsi).ab,ti.) AND (exp "automation"/ OR exp "electronics"/ OR "Software"/ OR "Computing Methodologies"/ OR exp "Computer Systems"/ OR "Automatic Data Processing"/ OR "Computational Biology"/ OR "Information Systems"/ OR "Hospital Information Systems"/ OR exp "Medical Informatics"/ OR Informatics/ OR exp "Medical Records Systems, Computerized"/ OR (automat* OR computer* OR electronic* OR software* OR data-mining OR ((surveillance) ADJ (tool*)) OR repositor* OR

information-system* OR informatics* OR bioinformatics* OR (electronic* ADJ3 record*)).ab,ti.) AND english.la.

Cochrane

((surveillance OR point-prevalence* OR incidence-rate*):ab,ti) AND ((((hospital* OR nosocomial* OR ward OR healthcare OR health-care OR blood-stream* OR bloodstream* OR surg* OR operative* OR postop* OR postsurg* OR catheter* OR ventilator* OR ICU OR NICU OR PICU OR intensive-care OR cross) NEAR/6 (infection*)) OR ((hospital* OR ventilator*) NEXT/1 (acquire* OR associat*) NEXT/1 (pneumon* OR event*)) OR hai OR hais OR hcai OR hcais OR ssi OR vap OR hap OR vaps OR haps OR clabsi):ab,ti) AND ((automat* OR computer* OR electronic* OR software* OR data-mining OR ((surveillance) NEXT/1 (tool*)) OR repositor* OR information-system* OR informatics* OR bioinformatics* OR (electronic* NEAR/3 record*)):ab,ti)

Web of science

TS=(((surveillance OR point-prevalence* OR incidence-rate*)) AND ((((hospital* OR nosocomial* OR ward OR healthcare OR health-care OR blood-stream* OR bloodstream* OR surg* OR operative* OR postop* OR postsurg* OR catheter* OR ventilator* OR ICU OR NICU OR PICU OR intensive-care OR cross) NEAR/5 (infection*)) OR ((hospital* OR ventilator*) NEAR/1 (acquire* OR associat*) NEAR/1 (pneumon* OR event*)) OR hai OR hais OR hcai OR hcais OR ssi OR vap OR hap OR vaps OR haps OR clabsi)) AND ((automat* OR computer* OR electronic* OR software* OR data-mining OR ((surveillance) NEAR/1 (tool*)) OR repositor* OR information-system* OR informatics* OR bioinformatics* OR (electronic* NEAR/2 record*)))) AND LA=(english)

Scopus

TITLE-ABS-KEY(((surveillance OR point-prevalence* OR incidence-rate*)) AND ((((hospital* OR nosocomial* OR ward OR healthcare OR health-care OR blood-stream* OR bloodstream* OR surg* OR operative* OR postop* OR postsurg* OR catheter* OR ventilator* OR ICU OR NICU OR PICU OR intensive-care OR cross) W/5 (infection*)) OR ((hospital* OR ventilator*) W/1 (acquire* OR associat*) W/1 (pneumon* OR event*)) OR hai OR hais OR hcai OR hcais OR ssi OR vap OR hap OR vaps OR haps OR clabsi)) AND ((automat* OR computer* OR electronic* OR software* OR data-mining OR ((surveillance) W/1 (tool*)) OR repositor* OR information-system* OR informatics* OR bioinformatics* OR (electronic* W/2 record*)))) AND LANGUAGE(english)

CINAHL (EBSCOhost)

(MH "Disease Surveillance" OR TI(surveillance OR point-prevalence* OR incidence-rate*)) AND (MH "Cross Infection+" OR MH "Surgical Wound Infection" OR MH "Catheter-Related Infections" OR TI(((hospital* OR nosocomial* OR ward OR healthcare OR health-care OR blood-stream* OR bloodstream* OR surg* OR operative* OR postop* OR postsurg* OR catheter* OR ventilator* OR ICU OR NICU OR PICU OR intensive-care OR cross) N5 (infection*)) OR ((hospital* OR ventilator*) N1 (acquire* OR associat*) N1 (pneumon* OR event*)) OR hai OR hais OR hcai OR hcais OR ssi OR vap OR hap OR vaps OR haps OR clabsi)) AND (MH "automation" OR MH "electronics" OR MH "Software" OR MH "Computing Methodologies" OR MH "Computer Systems" OR MH "Bioinformatics" OR MH "Information Systems" OR MH "Hospital Information Systems" OR MH "Medical Informatics" OR MH Informatics OR MH "Computerized Patient Record" OR TI(automat* OR computer* OR electronic* OR software* OR data-mining OR ((surveillance) N1 (tool*)) OR repositor* OR information-system* OR informatics* OR bioinformatics* OR (electronic* N2 record*))) AND LA(english)

Google scholar

First 100: Surveillance "hospital|nosocomial|bloodstream|surgical|catheter|ventilator infections"|"hospital|bloodstream|surgical|catheter|ventilator * infections" automation|computerized|computer|electronic|software|"data mining"
First 50: allintitle: "hospital|nosocomial|bloodstream|surgical|catheter|ventilator infections"|"hospital|bloodstream|surgical|catheter|ventilator * infections" automation|computerized|computer|electronic|software|"data mining"

Table 1. Description of the 78 studies included in the review, chronologically ordered and stating the variables used in their algorithms (see table 1 in the main text for explanation of the different categories). The quality indicators, as described in the article 'Electronically Assisted Surveillance of Healthcare Associated Infections' table 2, yielded 'red' boxes in case of low quality for the given indicator, 'orange' boxes for intermediate quality and 'green' boxes for good quality.

**Reference**	**Author**	**Continent**	**Year**	**Algorithm category**	**Quality indicators (see table 2 in the main article)**					
					1	**2**	**3**	**4**	**5**	**6**
[4]	Evans	North-America	1986	Microbiology +Antibiotics	Orange	Green	Green	Green	Red	Green
[5]	Broderick	North-America	1990	Microbiology +Antibiotics	Orange	Green	Green	Green	Orange	Orange
[6]	Evans	North-America	1992	Other	Green	Green	Green	Green	Orange	Red
[7]	Hirschhorn	North-America	1993	Other	Orange	Green	Red	Green	Green	Red
[8]	Rocha	North-America	1994	Microbiology +Antibiotics	Green	Red	Red	Green	Orange	Red
[9]	Baker	North-America	1995	Other	Red	Green	Red	Red	Green	Orange
[10]	Bouam	Europe	2003	Micro	Orange	Red	Orange	Orange	Green	Green
[11]	Graham	North-America	2004	Micro	Green	Red	Red	Red	Green	Red
[12]	Moro	Europe	2004	ICD	Red	Green	Red	Red	Red	Red
[13]	Trick	North-America	2004	Micro	Orange	Red	Green	Red	Orange	Red
[14]	Chalfine	Europe	2005	Micro	Orange	Green	Red	Red	Orange	Red
[15]	Haas	North-America	2005	Other	Red	Red	Red	Red	Green	Red
[16]	Mendonca	North-America	2005	Other	Red	Green	Red	Red	Green	Red
[17]	Brossette	North-America	2006	Micro	Orange	Red	Green	Orange	Orange	Green
[18]	Leth	Europe	2006	Other	Green	Green	Orange	Green	Green	Red
[19]	Pokorny	Europe	2006	Other	Orange	Green	Red	Green	Orange	Red
[20]	Bellini	Europe	2007	Micro	Green	Red	Orange	Orange	Orange	Red
[21]	Klompas	North-America	2008	Other	Red	Green	Red	Red	Orange	Red
[22]	Woeltje	North-America	2008	Microbiology +Antibiotics	Red	Red	Orange	Red	Green	Red
[23]	Bolon	North-America	2009	Other	Orange	Green	Red	Red	Red	Orange
[24]	Brown	Europe	2009	Other	Orange	Green	Green	Green	Orange	Green
[25]	Claridge	North-America	2009	Other	Orange	Green	Red	Red	Orange	Red
[26]	Bearman	North-America	2010	Other	Green	Green	Green	Orange	Green	Red
[27]	Leth	Europe	2010	Other	Orange	Green	Orange	Red	Green	Red
[28]	Apte	North-America	2011	ICD	Red	Green	Red	Red	Red	Red
[29]	Blacky	Europe	2011	Other	Red	Red	Red	Green	Green	Green
[30]	Bouzbid	Europe	2011	Other	Red	Green	Red	Orange	Green	Orange
[31]	Chang	Asia	2011	Other	Green	Red	Green	Green	Green	Red

Reference	Author	Continent	Year	Algorithm category	Quality indicators (see table 2 in the main article)					
					1	2	3	4	5	6
[32]	Choudhuri	North-America	2011	Other						
[33]	Hollenbeak	North-America	2011	Micro						
[34]	Inacio	North-America	2011	ICD						
[35]	van Mourik	Europe	2011	Microbiology +Antibiotics +Chemistry						
[36]	Woeltje	North-America	2011	Microbiology +Antibiotics						
[37]	Gerbier-Colomban	Europe	2012	Other						
[38]	Stamm	North-America	2012	Micro						
[39]	van Mourik	Europe	2012	Microbiology +Antibiotics +Chemistry						
[40]	De Bruin	Europe	2013	Other						
[41]	Hautemanière	Europe	2013	Other						
[42]	Henry	North-America	2013	Other						
[43]	Knepper	North-America	2013	Other						
[44]	Lo	Asia	2013	Microbiology +Antibiotics						
[45]	Tseng	Asia	2013	Other						
[46]	van Mourik	Europe	2013	ICD						
[47]	Venable	North-America	2013	Micro						
[48]	Kaiser	Europe	2014	Other						
[49]	Klouwenberg	Europe	2014	Other						
[50]	Branch-Elliman	North-America	2014	Other						
[51]	De Bus	Europe	2014	Other						
[52]	Du	Asia	2014	Other						
[53]	King	Europe	2014	Other						
[54]	Knepper	North-America	2014	Other						
[55]	Leclere	Europe	2014	Other						
[56]	Michelson	North-America	2014	Other						
[57]	Stevens	North-America	2014	Other						
[58]	Streefkerk	Europe	2014	Microbiology +Antibiotics +Chemistry						
[59]	Streefkerk	Europe	2014	Microbiology +Antibiotics +Chemistry						
[60]	Wald	North-America	2014	Other						
[61]	Yu	Asia	2014	ICD						
[62]	Tanushi	Europe	2014	Other						

Reference	Author	Continent	Year	Algorithm category	Quality indicators (see table 2 in the main article)					
					1	2	3	4	5	6
[63]	Branch-Elliman	North-America	2015	Other						
[64]	Hsu	North-America	2015	Microbiology +Antibiotics +Chemistry						
[65]	Mann	North-America	2015	Other						
[66]	Nuckchady	North-America	2015	Other						
[67]	Redder	Europe	2015	Microbiology +Antibiotics						
[68]	Tseng	Asia	2015	Other						
[69]	van Mourik	Europe	2015	Microbiology +Antibiotics +Chemistry						
[70]	Kulaylat	North-America	2016	Micro						
[71]	Ridgway	North-America	2016	Micro						
[72]	Perdiz	South-America	2016	Other						
[73]	Condell	Europe	2016	Other						
[74]	Bond	North-America	2016	ICD						
[75]	Leal	North-America	2016	Micro						
[76]	Streefkerk	Europe	2016	Other						
[77]	Marra	North-America	2017	ICD						
[78]	Gubbels	Europe	2017	Micro						
[79]	Sips	Europe	2017	Microbiology +Antibiotics						
[80]	Hebert	North-America	2018	Microbiology +Antibiotics +Chemistry						
[81]	Pindyck	North-America	2018	Other						

References

1. Liberati, A., The PRISMA Statement for Reporting Systematic Reviews and Meta-Analyses of Studies That Evaluate Health Care Interventions: Explanation and Elaboration. BMJ, 2009. 339:b2700.
2. Bramer, W.M., *et al*., A systematic approach to searching: an efficient and complete method to develop literature searches. J Med Libr Assoc, 2018. 106(4): p. 531-541.
3. Bramer, W.M., Reference checking for systematic reviews using Endnote. J Med Libr Assoc, 2018. 106(4): p. 542-546.
4. Evans, R.S., R.A. Larsen, and J.P. Burke, Computer surveillance of hospital-acquired infections and antibiotic use. J AM MED ASSOC, 1986. 256(8): p. 1007-1011.
5. Broderick, A., *et al*., Nosocomial infections: Validation of surveillance and computer modeling to identify patients at risk. AM J EPIDEMIOL, 1990. 131(4): p. 734-742.
6. Evans, R.S., *et al*., Computerized identification of patients at high risk for hospital-acquired infection. AM J INFECT CONTROL, 1992. 20(1): p. 4-10.
7. Hirschhorn, L.R., J.S. Currier, and R. Platt, Electronic surveillance of antibiotic exposure and coded discharge diagnoses as indicators of postoperative infection and other quality assurance measures. Infect Control Hosp Epidemiol, 1993. 14(1): p. 21-8.
8. Rocha, B.H., *et al*., Computerized detection of nosocomial infections in newborns. Proc Annu Symp Comput Appl Med Care, 1994: p. 684-8.

9. Baker, C., *et al*., Comparison of case-finding methodologies for endometritis after cesarean section. Am J Infect Control, 1995. 23(1): p. 27-33.
10. Bouam, S., *et al*., An intranet-based automated system for the surveillance of nosocomial infections: prospective validation compared with physicians' self-reports. Infect Control Hosp Epidemiol, 2003. 24(1): p. 51-5.
11. Graham Iii, P.L., *et al*., Validation of a multicenter computer-based surveillance system for hospital-acquired bloodstream infections in neonatal intensive care departments. Am J Infect Control, 2004. 32(4): p. 232-234.
12. Moro, M.L. and F. Morsillo, Can hospital discharge diagnoses be used for surveillance of surgical-site infections? 2004. 56(3): p. 239-241.
13. Trick, W.E., *et al*., Computer algorithms to detect bloodstream infections. Emerg Infect Dis, 2004. 10(9): p. 1612-1620.
14. Chalfine, A., *et al*., Highly sensitive and efficient computer-assisted system for routine surveillance for surgical site infection. Infect Control Hosp Epidemiol, 2006. 27(8): p. 794-801.
15. Haas, J.P., *et al*., Use of computerized surveillance to detect nosocomial pneumonia in neonatal intensive care unit patients. Am J Infect Control, 2005. 33(8): p. 439-443.
16. Mendonca, E.A., *et al*., Extracting information on pneumonia in infants using natural language processing of radiology reports. J. Biomed. Inform., 2005. 38(4): p. 314-321.
17. Brossette, S.E., *et al*., A laboratory-based, hospital-wide, electronic marker for nosocomial infection: the future of infection control surveillance? Am J Clin Pathol, 2006. 125(1): p. 34-9.
18. Leth, R.A. and J.K. Møller, Surveillance of hospital-acquired infections based on electronic hospital registries. J Hosp Infect, 2006. 62(1): p. 71-79.
19. Pokorny, L., *et al*., Automatic detection of patients with nosocomial infection by a computer-based surveillance system: a validation study in a general hospital. Infect Control Hosp Epidemiol, 2006. 27(5): p. 500-3.
20. Bellini, C., *et al*., Comparison of automated strategies for surveillance of nosocomial bacteremia. Infect Control Hosp Epidemiol, 2007. 28(9): p. 1030-5.
21. Klompas, M., K. Kleinman, and R. Platt, Development of an algorithm for surveillance of ventilator-associated pneumonia with electronic data and comparison of algorithm results with clinician diagnoses. Infect Control Hosp Epidemiol, 2008. 29(1): p. 31-7.
22. Woeltje, K.F., *et al*., Automated surveillance for central line-associated bloodstream infection in intensive care units. Infect Control Hosp Epidemiol, 2008. 29(9): p. 842-6.
23. Bolon, M.K., *et al*., Improved surveillance for surgical site infections after orthopedic implantation procedures: Extending applications for automated data. Clin Infect Dis, 2009. 48(9): p. 1223-1229.
24. Brown, C., *et al*., Use of anti-infective serial prevalence studies to identify and monitor hospital-acquired infection. J Hosp Infect, 2009. 73(1): p. 34-40.
25. Claridge, J.A., *et al*., Who is monitoring your infections: shouldn't you be? Surg Infect (Larchmt), 2009. 10(1): p. 59-64.
26. Bearman, G.M.L. and M.I. Oppenheim, A Clinical Predictive Model for Catheter Related Bloodstream Infections from the Electronic Medical Record. Open Epidemiology …. 2010: benthamopen.com.
27. Leth, R.A., *et al*., Surveillance of selected post-caesarean infections based on electronic registries: Validation study including post-discharge infections. J Hosp Infect, 2010. 75(3): p. 200-204.
28. Apte, M., *et al*., Comparison of two computer algorithms to identify surgical site infections. Surg Infect, 2011. 12(6): p. 459-464.
29. Blacky, A., *et al*., Fully Automated Surveillance of Healthcare-Associated Infections with MONI-ICU: A Breakthrough in Clinical Infection Surveillance. 2011. 2(3): p. 365-372.
30. Bouzbid, S., *et al*., Automated detection of nosocomial infections: Evaluation of different strategies in an intensive care unit 2000-2006. J Hosp Infect, 2011. 79(1): p. 38-43.
31. Chang, Y.J., *et al*., Predicting hospital-acquired infections by scoring system with simple parameters. PLoS One, 2011. 6(8): p. e23137.
32. Choudhuri, J.A., *et al*., An electronic catheter-associated urinary tract infection surveillance tool. Infect Control Hosp Epidemiol, 2011. 32(8): p. 757-762.
33. Hollenbeak, C.S., *et al*., Electronic measures of surgical site infection: implications for estimating risks and costs. Infect Control Hosp Epidemiol, 2011. 32(8): p. 784-90.
34. Inacio, M.C., *et al*., Leveraging electronic medical records for surveillance of surgical site infection in a total joint replacement population. Infect Control Hosp Epidemiol, 2011. 32(4): p. 351-9.

35. van Mourik, M.S., *et al*., Automated detection of external ventricular and lumbar drain-related meningitis using laboratory and microbiology results and medication data. PLoS ONE, 2011. 6(8): p. e22846.
36. Woeltje, K.F., *et al*., Electronic surveillance for healthcare-associated central line-associated bloodstream infections outside the intensive care unit. Infect Control Hosp Epidemiol, 2011. 32(11): p. 1086-90.
37. Gerbier-Colomban, S., *et al*., Evaluation study of different strategies for detecting surgical site infections using the hospital information system at Lyon University Hospital, France. Ann Surg, 2012. 255(5): p. 896-900.
38. Stamm, A.M. and C.J. Bettacchi, A comparison of 3 metrics to identify health care-associated infections. Am J Infect Control, 2012. 40(8): p. 688-691.
39. van Mourik, M.S.M., *et al*., Automated Detection of Healthcare Associated Infections: External Validation and Updating of a Model for Surveillance of Drain-Related Meningitis. PLoS ONE, 2012. 7(12).
40. De Bruin, J.S., *et al*., Effectiveness of an automated surveillance system for intensive care unit-acquired infections. J Am Med Informatics Assoc, 2013. 20(2): p. 369-372.
41. Hautemanière, A., *et al*., Screening for surgical nosocomial infections by crossing databases. J Infect Public Health, 2013. 6(2): p. 89-97.
42. Henry, F.F., *et al*., Exploring the frontier of electronic health record surveillance the case of postoperative complications. Med Care, 2013. 51(6): p. 509-516.
43. Knepper, B.C., *et al*., Time-saving impact of an algorithm to identify potential surgical site infections. Infect Control Hosp Epidemiol, 2013. 34(10): p. 1094-1098.
44. Lo, Y.S., W.S. Lee, and C.T. Liu, Utilization of electronic medical records to build a detection model for surveillance of healthcare-associated urinary tract infections. J Med Syst, 2013. 37(2).
45. Tseng, Y.J., *et al*., Rule-based healthcare-associated bloodstream infection classification and surveillance system. Stud Health Technol Inform, 2013. 186: p. 145-9.
46. van Mourik, M.S., *et al*., Accuracy of hospital discharge coding data for the surveillance of drain-related meningitis. 2013. 34(4): p. 433-436.
47. Venable, A. and S. Dissanaike, Is automated electronic surveillance for healthcare-associated infections accurate in the burn unit? J Burn Care Res, 2013. 34(6): p. 591-597.
48. Kaiser, A.M., *et al*., Development of trigger-based semi-automated surveillance of ventilator-associated pneumonia and central line-associated bloodstream infections in a Dutch intensive care. Ann Intensive Care, 2014. 4(1).
49. Klouwenberg, P.M.C.K., *et al*., Electronic implementation of a novel surveillance paradigm for ventilator-associated events feasibility and validation. Am J Respir Crit Care Med, 2014. 189(8): p. 947-955.
50. Branch-Elliman, W., *et al*., Using clinical variables to guide surgical site infection detection: A novel surveillance strategy. Am J Infect Control, 2014. 42(12): p. 1291-1295.
51. De Bus, L., *et al*., Validity analysis of a unique infection surveillance system in the intensive care unit by analysis of a data warehouse built through a workflow-integrated software application. J Hosp Infect, 2014. 87(3): p. 159-164.
52. Du, M., *et al*., Real-time automatic hospital-wide surveillance of nosocomial infections and outbreaks in a large Chinese tertiary hospital. BMC Med Inform Decis Mak, 2014. 14: p. 9.
53. King, C., *et al*., Syndromic surveillance of surgical site infections - A case study in coronary artery bypass graft patients. J. Infect., 2014. 68(1): p. 23-31.
54. Knepper, B.C., *et al*., Identifying colon and open reduction of fracture surgical site infections using a partially automated electronic algorithm. Am J Infect Control, 2014. 42(10): p. S291-S295.
55. Leclère, B., *et al*., Matching bacteriological and medico-administrative databases is efficient for a computer-enhanced surveillance of surgical site infections: Retrospective analysis of 4,400 surgical procedures in a French university hospital. Infect Control Hosp Epidemiol, 2014. 35(11): p. 1330-1335.
56. Michelson, J.D., J.S. Pariseau, and W.C. Paganelli, Assessing surgical site infection risk factors using electronic medical records and text mining. Am J Infect Control, 2014. 42(3): p. 333-336.
57. Stevens, J.P., *et al*., Automated surveillance for ventilator-associated events. Chest, 2014. 146(6): p. 1612-1618.

58. Streefkerk, R.H.R.A., *et al*., Evaluation of an algorithm for electronic surveillance of hospital-acquired infections yielding serial weekly point prevalence scores. Infect Control Hosp Epidemiol, 2014. 35(7): p. 888-890.
59. Streefkerk, R.H.R.A., *et al*., An automated algorithm to preselect patients to be assessed individually in point prevalence surveys for hospital-acquired infections in surgery. Infect Control Hosp Epidemiol, 2014. 35(7): p. 886-887.
60. Wald, H.L., *et al*., Accuracy of electronic surveillance of catheter-associated urinary tract infection at an academic medical center. Infect Control Hosp Epidemiol, 2014. 35(6): p. 685-691.
61. Yu, T.H., *et al*., Is it possible to identify cases of coronary artery bypass graft postoperative surgical site infection accurately from claims data? BMC Med. Inform. Decis. Mak., 2014. 14.
62. Tanushi, H., M. Kvist, and E. Sparrelid, Detection of healthcare-associated urinary tract infection in Swedish electronic health records. 2014. 207: p. 330-339.
63. Branch-Elliman, W., *et al*., Natural language processing for real-time catheter-associated urinary tract infection surveillance: Results of a pilot implementation trial. Infect Control Hosp Epidemiol, 2015. 36(9): p. 1004-1010.
64. Hsu, H.E., *et al*., An electronic surveillance tool for catheter-associated urinary tract infection in intensive care units. Am J Infect Control, 2015. 43(6): p. 592-599.
65. Mann, T., *et al*., Building and validating a computerized algorithm for surveillance of ventilator-associated events. Infect Control Hosp Epidemiol, 2015. 36(9): p. 999-1003.
66. Nuckchady, D., *et al*., Assessment of an automated surveillance system for detection of initial ventilator-associated events. Am J Infect Control, 2015. 43(10): p. 1119-1121.
67. Redder, J.D., R.A. Leth, and J.K. Møller, Incidence rates of hospital-acquired urinary tract and bloodstream infections generated by automated compilation of electronically available healthcare data. J Hosp Infect, 2015. 91(3): p. 231-236.
68. Tseng, Y.J., *et al*., A Web-Based, Hospital-Wide Health Care-Associated Bloodstream Infection Surveillance and Classification System: Development and Evaluation. JMIR Med Inform, 2015. 3(3): p. e31.
69. Van Mourik, M.S.M., *et al*., Validation of an automated surveillance approach for drain-related meningitis: A multicenter study. Infect Control Hosp Epidemiol, 2015. 36(1): p. 65-75.
70. Kulaylat, A.N., *et al*., Measuring Surgical Site Infections in Children: Comparing Clinical, Electronic, and Administrative Data. J Am Coll Surg, 2016. 222(5): p. 823-30.
71. Ridgway, J.P., *et al*., Performance characteristics and associated outcomes for an automated surveillance tool for bloodstream infection. Am J Infect Control, 2016.
72. Perdiz, L.B., *et al*., Impact of an Automated Surveillance to Detect Surgical-Site Infections in Patients Undergoing Total Hip and Knee Arthroplasty in Brazil. Infect Control Hosp Epidemiol, 2016: p. 1-3.
73. Condell, O., *et al*., Automated surveillance system for hospital-acquired urinary tract infections in Denmark. J Hosp Infect, 2016. 93(3): p. 290-296.
74. Bond, J., *et al*., Comparing administrative and clinical data for central line associated blood stream infections in Pediatric Intensive Care Unit and Pediatric Cardiothoracic Intensive Care Unit. Infect. Dis. Rep., 2016. 8(3): p. 58-62.
75. Leal, J.R., *et al*., The Validation of a Novel Surveillance System for Monitoring Bloodstream Infections in the Calgary Zone. Can J Infect Dis Med Microbiol, 2016. 2016.
76. Streefkerk, H.R.A., *et al*., Internal and External Validation of a Computer-Assisted Surveillance System for Hospital-Acquired Infections in a 754-Bed General Hospital in the Netherlands. Infect Control Hosp Epidemiol, 2016. 37(11): p. 1355-1360.
77. Marra, A.R., M. Alkatheri, and M.B. Edmond, Catheter-Associated Urinary Tract Infection: Utility of the ICD-10 Metric as a Surrogate for the National Healthcare Safety Network (NHSN) Surveillance Metric. Infect Control Hosp Epidemiol, 2017. 38(4): p. 506-507.
78. Gubbels, S., *et al*., National automated surveillance of hospital-acquired bacteremia in Denmark using a computer algorithm. Infect Control Hosp Epidemiol, 2017. 38(5): p. 559-566.
79. Sips, M.E., M.J.M. Bonten, and M.S.M. Van Mourik, Semiautomated Surveillance of Deep Surgical Site Infections After Primary Total Hip or Knee Arthroplasty. Infect Control Hosp Epidemiol, 2017. 38(6): p. 732-735.
80. Hebert, C., *et al*., Development and validation of an automated ventilator-associated event electronic surveillance system: A report of a successful implementation. Am J Infect Control, 2017.

81. Pindyck, T., *et al*., Validation of an electronic tool for flagging surgical site infections based on clinical practice patterns for triaging surveillance: Operational successes and barriers. Am J Infect Control, 2017.
